# Intrapericardial Delivery of Gelfoam Enables the Targeted Delivery of Periostin Peptide after Myocardial Infarction by Inducing Fibrin Clot Formation

**DOI:** 10.1371/journal.pone.0036788

**Published:** 2012-05-10

**Authors:** Brian D. Polizzotti, Shima Arab, Bernhard Kühn

**Affiliations:** 1 Department of Cardiology, Children's Hospital Boston, Boston, Massachusetts, United States of America; 2 Department of Pediatrics, Harvard Medical School, Boston, Massachusetts, United States of America; Foundation for Applied Medical Research, Spain

## Abstract

**Background:**

Administration of a recombinant peptide of Periostin (rPN) has recently been shown to stimulate cardiomyocyte proliferation and angiogensis after myocardial infarction (MI) [1]. However, strategies for targeting the delivery of rPN to the heart are lacking. Intrapericardial administration of drug-eluting hydrogels may provide a clinically viable strategy for increasing myocardial retention, therapeutic efficacy, and bioactivity of rPN and to decrease systemic re-circulation.

**Methods and Results:**

We investigated the ability of intrapericardial injections of drug-eluting hydrogels to deliver and prolong the release of rPN to the myocardium in a large animal model of myocardial infarction. Gelfoam is an FDA-approved hemostatic material commonly used in surgery, and is known to stimulate fibrin clot formation. We show that Gelfoam disks loaded with rPN, when implanted within the pericardium or peritoneum of mammals becomes encapsulated within a non-fibrotic fibrin-rich hydrogel, prolonging the *in vitro* and *in vivo* release of rPN. Administration into the pericardial cavity of pigs, following a complete occlusion of the left anterior descending artery, leads to greater induction of cardiomyocyte mitosis, increased cardiomyocyte cell cycle activity, and enhanced angiogenesis compared to direct injection of rPN alone.

**Conclusions:**

The results of this study suggest that intrapericardial drug delivery of Gelfoam, enhanced by triggered clot formation, can be used to effectively deliver rPN to the myocardium in a clinically relevant model of myocardial infarction. The work presented here should enhance the translational potential of pharmaceutical-based strategies that must be targeted to the myocardium.

## Introduction

Myocardial infarction (MI) results in the irreversible damage of cardiac muscle, which ultimately leads to heart failure. Current clinical strategies are mainly focused on attenuating the progressive deterioration of the remaining viable myocardium [2]. Recently, however, there has been a paradigm shift toward strategies aimed at regenerating the myocardium to improve cardiac function [1], [3], [4]. One such approach involves the administration of pharmaceuticals capable of either replacing lost cardiomyocytes by stimulating recruitment and differentiation of resident cardiac stem cells [5] or by enhancing the proliferation of endogenous cardiomyocytes [1], [3].

We have recently demonstrated that administration of a recombinant truncated peptide of periostin (referred to as rPN throughout this manuscript) in rats after MI increased cardiomyocyte mitosis, improved ventricular remodeling and myocardial function, reduced fibrosis and infarct size, and increased angiogenesis [1]. Full length periostin is a matricellular protein that consists of an N-terminal domain, tandem repeats of four fasciclin I domains, and an alternatively spliced C-terminal region [6]. In the developing heart, periostin is expressed in the atrioventricular cushions and in the myocardium. However, in the adult heart, expression is reduced to very low levels [6]–[12], but is re-expressed after MI, where it plays a role in the maturation of the infarct scar [8], [9].

Use of rPN for the treatment of heart disease in humans would require a system that permits the effective and targeted delivery to the myocardium. The most commonly employed strategies for delivering factors to the heart, which includes systemic, intracoronary, and intramyocardial injections, are of limited utility due to the low solubility of rPN at therapeutically relevant concentrations. In addition, without a tailored delivery vehicle, these approaches have very poor specificity for the myocardium, necessitating relatively large doses and increasing the potential for adverse reactions in neighboring normal tissue.

Alternate, clinically relevant strategies that enable targeted delivery to the heart and that minimize systemic circulation are drastically needed. One such approach is the implantation of drug-eluting devices into the pericardial cavity (i.e. the virtual space between the parietal pericardium and visceral layer). Previous studies in chronically ischemic pigs have shown that direct intrapericardial injection of growth factors resulted in increased myocardial deposition and retention and decreased systemic recirculation, compared to intracoronary or intravenous delivery [13]. Implantation of a drug-eluting device within the pericardial cavity might offer several advantages to direct injections, including sustained spatio-temporal delivery of multiple factors over multiple time scales, reduced proteolytic degradation (especially after MI), and enhanced myocardial retention [14].

Gelfoam is an FDA-approved hemostatic medical device prepared from purified pork skin gelatin USP granules and crosslinked by a thermal treatment method [15]. It is water-insoluble, bioresorbable, inexpensive, non-allergenic, and is able to absorb and hold within its interstices many times its weight of blood and other fluids. Gelfoam can be milled and easily injected through a 5Fr catheter, which is necessary for intrapericardial injections. Recently, Gelfoam disks loaded with mesenchymal stem cells were injected into the pericardial cavity of pigs [16]. The procedure was performed using minimally invasive techniques, and the presence of Gelfoam in the pericardial cavity was not associated with cardiac effusions or adhesions, suggesting that this approach is clinically viable. Gelfoam disks have also been utilized as a drug delivery vehicle [15], [17]–[21]. However, the use of Gelfoam to deliver therapeutics has been hindered by poor release kinetics [15], [17]–[21]. The addition of retardants such as polyethylene glycol 400 monostearate, cetyl ester wax, and collagen hydrogels have been shown to prolong the drug release [15], [22]. Interestingly, Gelfoam stimulates the clotting cascade in which fibrin becomes deposited within the intersitital pores of the disk. We hypothesized that intrapericardial injection of Gelfoam, loaded with rPN, into the pericardial cavity would stimulate spontaneous *in vivo* fibrin-hydrogel formation and enable the prolonged release of rPN to the myocardium following MI.

In the present paper, we characterize the *in vitro* and *in vivo* drug delivery of rPN from Gelfoam disks. We demonstrate that Gelfoam disks become encased within fibrin-rich hydrogels when implanted in the pericardial or peritoneal fluid of pigs or mice, respectively. We show that Gelfoam-induced fibrin hydrogels enable the prolonged release of rPN *in vitro* and within the peritoneal cavity of mice, *in vivo*. We also demonstrate that administration of Gelfoam disks loaded with rPN into the pericardial cavity of pigs, following a complete occlusion of the left anterior descending artery, leads to increased cardiomyocyte cell cycle activity and angiogenesis, compared to direct injection of rPN alone.

## Results

### Gelfoam disks become encapsulated within a fibrin matrix after peritoneal implantation in mice

**Figure 1:**
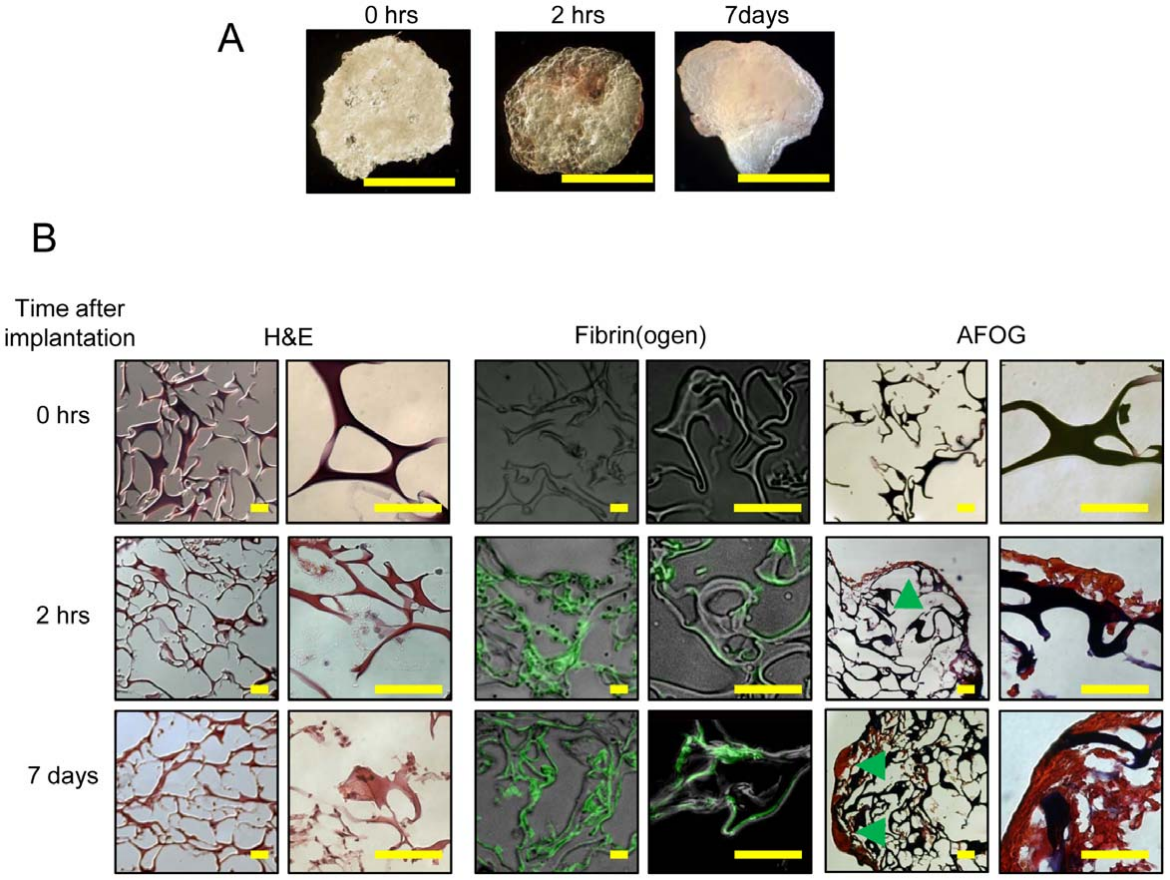
Gelfoam hydrogels explanted from the peritoneal cavity of mice are encased within a fibrin matrix. (A) Photomicrographs of explanted Gelfoam sponges at various time points. (B) Explanted Gelfoam sponges were characterized by immunohistochemistry and immunofluorescence. Sections were stained with hemotoxylin and eosin (H&E), fibrin(ogen), and acid fuschin orange –G (AFOG). Green arrows indicate fibrin deposition within the Gelfoam sponge. Scale bars, (A) 10 mm; (B) 50 µm.

We hypothesized that Gelfoam's innate ability to stimulate *in vivo* clot formation would enable the prolonged release of rPN when administered intrapericardially. To test whether Gelfoam disks become encapsulated within fibrin hydrogels in the absence of severe bleeding, we implanted pre-swollen Gelfoam disks (in PBS) into the peritoneum of mice. Animals were sacrificed after 2 hrs and 7 days, the devices were recovered, and histological analysis was performed (**Figure 1A**). Hematoxylin and eosin (H&E) staining revealed that Gelfoam induced a minimal inflammatory response at both time points (**Figure 1B**), which is in agreement with previously reported studies [23]–[26]. Acid Fuchsin Orange-G (AFOG) staining revealed the presence of a dense fibrin network that interpenetrated the Gelfoam porous network (**Figure 1B**). The presence of fibrin was confirmed by immunofluorecent staining on sections with an antibody against fibrin(ogen) (**Figure 1B**). It is important to note that Gelfoam disks were not encased within a fibrotic capsule (lack of blue staining in AFOG), which is in agreement with previous studies [24]–[26] and suggests that Gelfoam disks do not induce a foreign body response.

### Fibrin prolongs rPN release from Gelfoam scaffolds In Vitro and In Vivo

**Figure 2:**
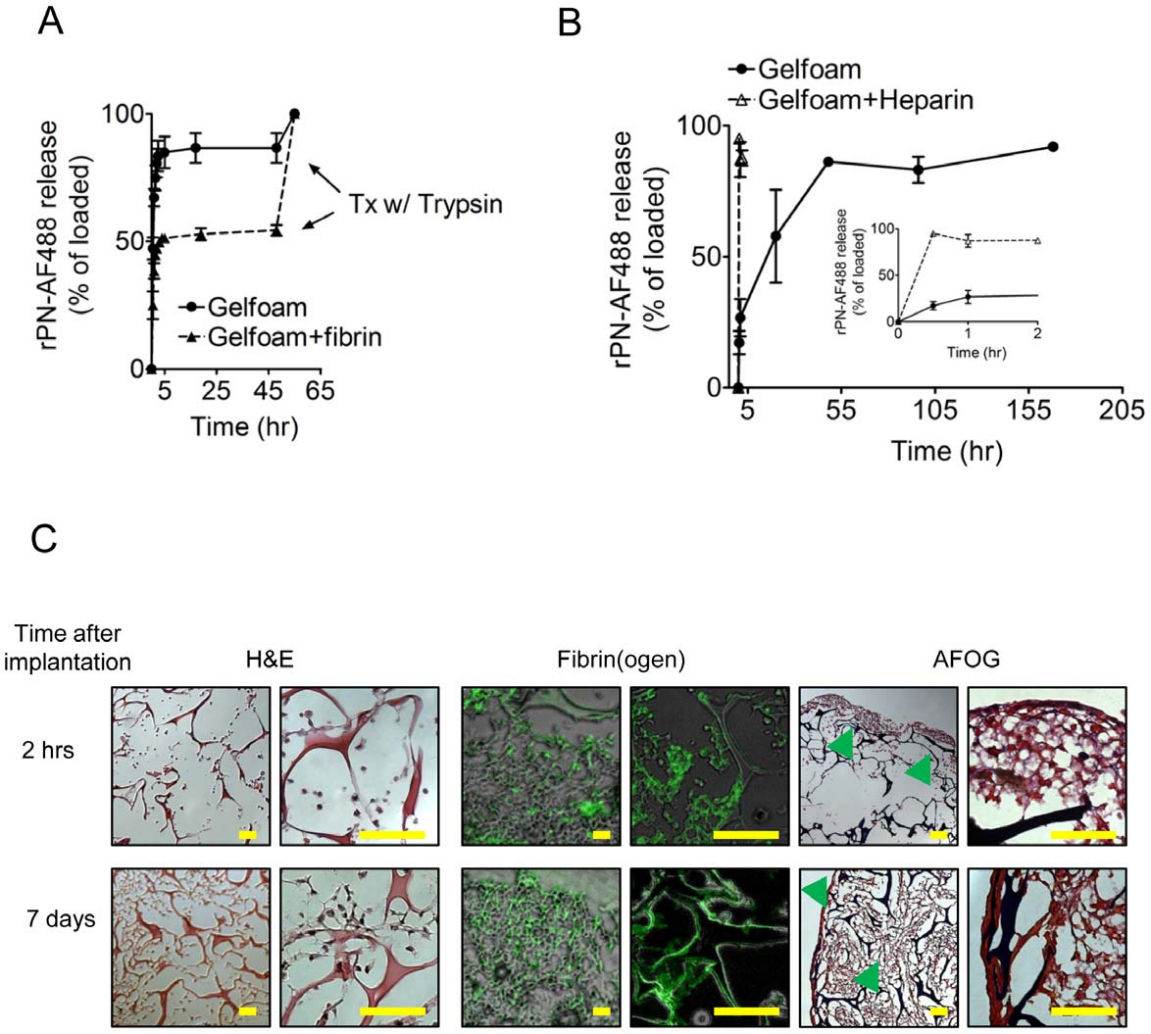
Fibrin encased Gelfoam hydrogels prolong rPN release In Vitro and In Vivo. (A) *In vitro* release kinetics of fluorescently-labeled rPN (rPN-AF488) from Gelfoam and Gelfoam-Fibrin composite hydrogels in PBS at 4°C. (B) *In vivo* release kinetics of rPN-AF488 from Gelfoam and Gelfoam/Heparin hydrogels implanted within the peritoneal cavity of mice. (C) Gelfoam sponges loaded with rPN –AF488 were explanted and characterized by immunohistochemistry and immunofluorescence. Sections were stained with hemotoxylin and eosin (H&E), fibrin(ogen), and acid fuschin orange –G (AFOG). Green arrows indicate fibrin deposition within the Gelfoam sponge. Scale bars, 50 µm.

To quantify the release of rPN from Gelfoam, we labeled rPN with the fluorophore Alexa Fluor 488 (**Figure S1**). The release profile of fluorescently-labeled rPN (rPN-AF488) from Gelfoam was assessed *in vitro* (**Figure 2A**). rPN-AF488 was rapidly released from Gelfoam disks. Interpolation of the release prolife indicates that the time required for 50 and 90% release (*t_50_* and *t_90_*) is ∼0.5 and ∼2 hrs, respectively (**Table 1**). After 2 days, the rate of rPN-AF488 release from Gelfoam remained constant, at which point we added trypsin to degrade the gels to confirm that all the rPN had been released from the Gelfoam. Interestingly, approximately 10% of the rPN initially loaded was still detected within the degraded gels, suggesting that either electrostatic and/or hydrophobic interactions may play a role in determining the release profile.

**Table 1:** Release Rates.

Gelfoam-rPN Device	t_50_ (hours)	t_90_ (hours)
*In vitro*	0.50	2.00
*In vivo*	15.00	50.00

In Vitro and In Vivo release rates from Gelfoam hydrogels.

We evaluated the ability of fibrin to prolong the release of rPN-AF488 from Gelfoam disks, *in vitro* (**Figure 2A**). To test this, we encapsulated Gelfoam disks loaded with rPN-AF488 within a fibrin hydrogel and incubated them in PBS at 4°C for 2 days. rPN-AF488 release from the composite hydrogels exhibited a smaller burst release compared to Gelfoam alone (25% vs 50% within the first 30 min, respectively) and a more sustained release of rPN-AF488 over the time period studied. These data suggest that Gelfoam's innate ability to induce fibrin deposition may provide a unique mechanism to prolong the *in vivo* release of rPN from Gelfoam matrices.

To evaluate the release profile *in vivo*, Gelfoam disks loaded with rPN-AF488 were implanted within the peritoneum of mice [27]. After 0.5, 2, 20, 48, 96, and 168 hrs, Gelfoam disks were retrieved and either degraded to determine the amount of rPN-AF488 released or processed for histological analysis. Following a minimal burst release within the first hour (∼26%), rPN displayed a sustained release from Gelfoam disks over the time period studied (**Figure 2B**). Interpolation from the graph indicates that the *t_50_* and *t_90_* values are 15 and 50 hrs, respectively (**Table 1**). It is important to note that these values are significantly higher than those obtained from the *in vitro* release experiments from Gelfoam alone (*t_50_* and *t_90_* values of 0.5 and 2 hrs, respectively; **Table 1**), indicating the fibrin is able to retard rPN release. Histological analysis of explanted disks, taken 2 hrs and 7 days post implantation, was characterized by mild inflammation and significant fibrin deposition within the interstitial pores of Gelfoam disks (**Figure 2C**).

To determine if fibrin deposition contributed to the observed release *in vivo*, we inhibited fibrin formation with heparin and performed similar release studies with Gelfoam disks preloaded with rPN-AF488 (**Figure 2B**, inset). Gelfoam disks treated with heparin rapidly released rPN-AF488 within 1 hour after implantation. This result is similar to the release rates of rPN-AF488 from Gelfoam disks alone *in vitro* and suggests that thrombosis formation plays a critical role in prolonging the release of rPN from Gelfoam matrices.

### rPN readily diffuses through damaged myocardium

**Figure 3:**
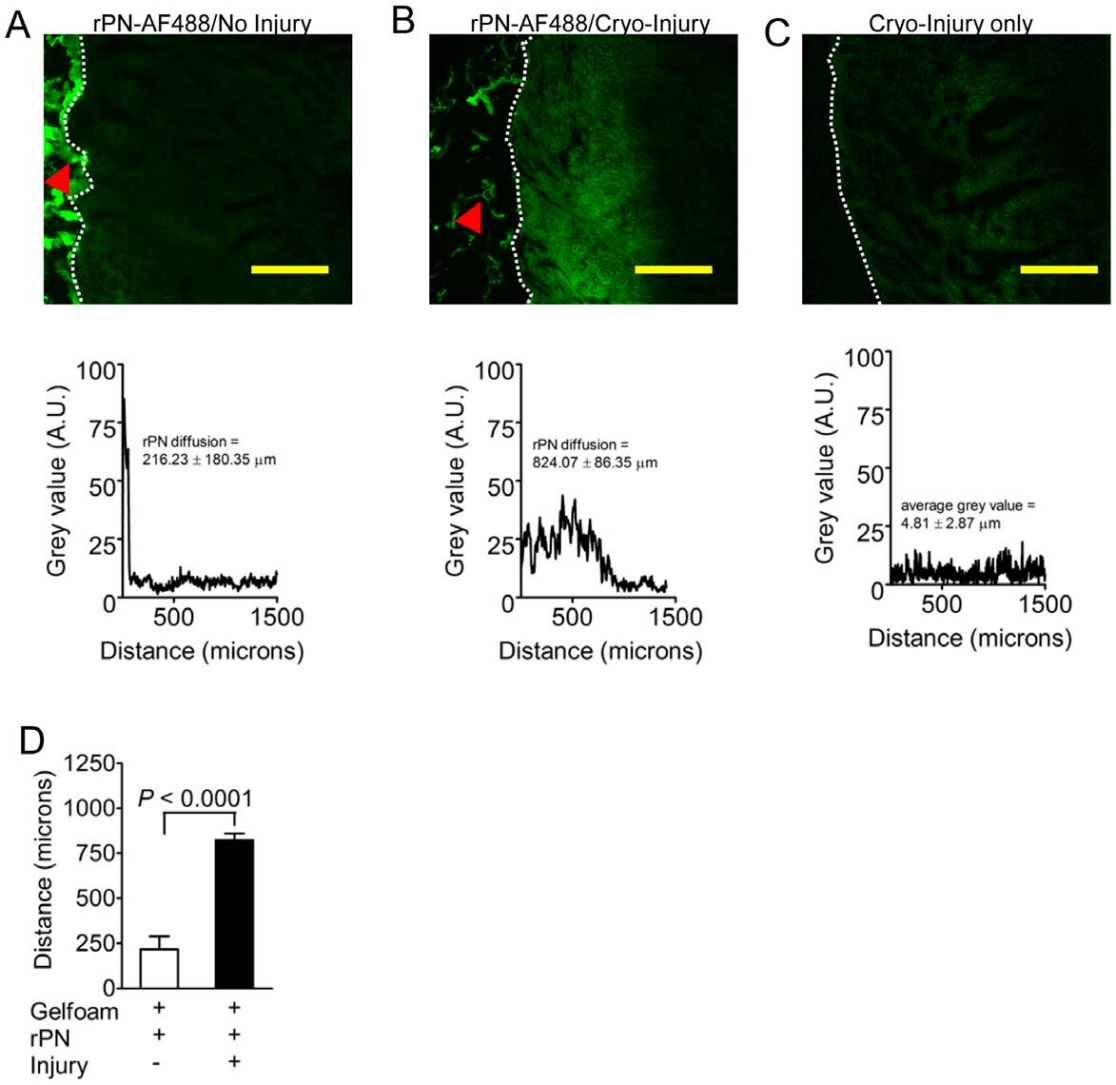
rPN readily diffuses into damaged myocardium. (A–B) Cross-sectional photomicrographs and pixel intensity profiles of rPN-AF488 diffusion through non-infarcted (A) and infarcted (B) mouse hearts. (C) Photomicrograph and pixel intensity prolife for cryo-injured heart only. (D) Quantification reveals that rPN readily diffuses through injured myocardium but not healthy intact muscle. Red arrows demarcate the epicardial surface and white arrows represent Gelfoam loaded with rPN-AF488. Analysis was performed 2 hrs after cryo-injury. The mean ± SEM of 6 independent pixel intensity profiles is provided in the graphs.

To determine if rPN can diffuse into the myocardium after intrapericardial injection we performed *ex vivo* diffusion experiments using mouse hearts. To evaluate the diffusibility of rPN before and after injury, fluorescently-labeled rPN was loaded onto Gelfoam disks, and glued to the left ventricular surface of normal and cryo-injured hearts. Subsequent histological analysis revealed that rPN diffusion through healthy myocardium was minimal (∼216 µm), compared to its diffusion through cryo-injured cardiac tissue (∼830 µm, *P*<0.0001, **Figure 3**) after 2 hours at 37°C and 5% CO_2_. These results suggest that intrapericardial administration is a highly effective method to deliver rPN specifically to the infarcted region of the myocardium.

### Gelfoam disks become encapsulated within a fibrin matrix after injection into the pericardial cavity of pigs

**Figure 4:**
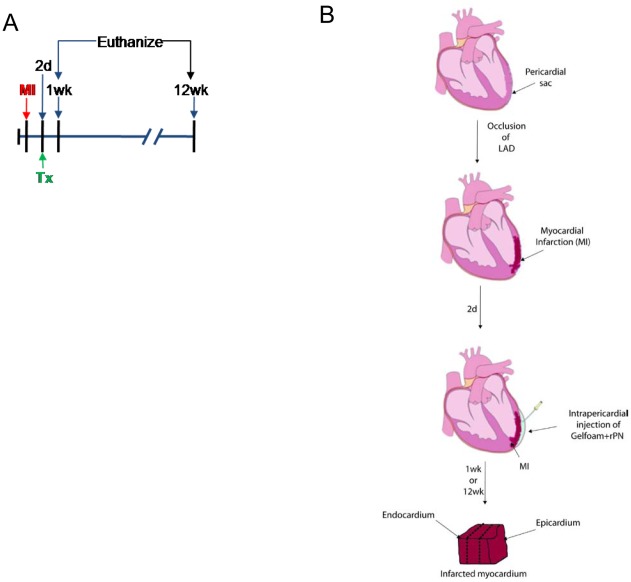
Myocardial infarction timeline and strategy for targeted delivery. (A) Experimental design for the delivery of rPN in a swine model of myocardial infarction (MI). (B) Schematic illustration of the intrapericardial delivery of Gelfoam/rPN system and our strategy for tissue collection.

**Figure 5:**
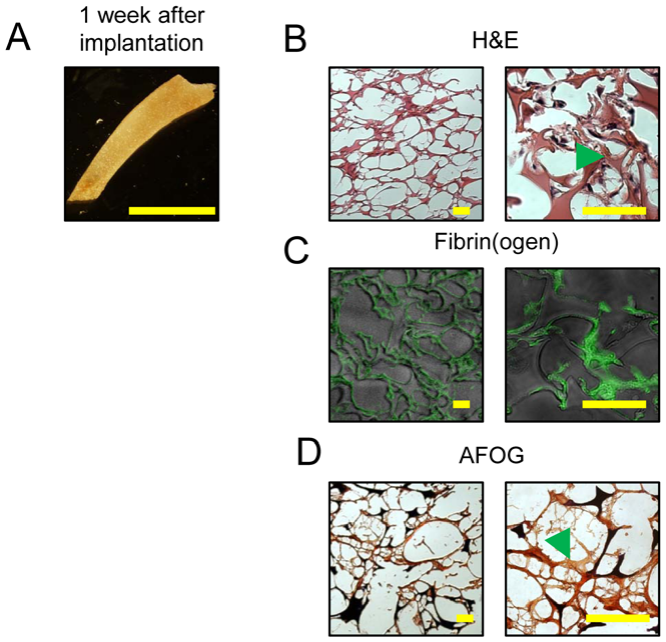
Gelfoam gels loaded with rPN become encased with fibrin-rich hydrogels after intrapericardial injection in pigs. (A) Cross-sectional photomicrograph of the explanted Gelfoam disk 7 days after implantation. (B–D) Explanted Gelfoam disks were characterize d with hematoxylin and eosin (H&E, B), immunofluorescent staining for fibrinogen (C) acid-fuschin orange-G (AFOG, D). Green arrows indicate fibrin deposition. Scale bars, (A) 20 mm; (B–D) 50 µm.

In order to verify that a similar clotting mechanism occurred in the pericardial cavity of large mammals, we injected Gelfoam loaded with or without rPN into the pericardial cavity of Yorkshire pigs following occlusion of the left anterior descending artery (**Figure 4**). One or twelve weeks later, animals were euthanized and a gross examination of the heart was conducted. One week after implantation, we observed a similar response as was seen when Gelfoam was implanted within the peritoneum of mice (**Figure 5A–D**). No evidence of the Gelfoam disk was present twelve weeks after implantation. To confirm that the material encasing the Gelfoam was similar to what we observed in the peritoneum of mice (i.e. fibrin), we stained sections with H&E (**Figure 5B**), AFOG (**Figure 5D**), and antibodies against fibin(ogen) (**Figure 5C**). As observed previously in the mouse peritoneum, implantation of Gelfoam within the pericardial cavity is characterized by a mild inflammatory response, significant fibrin deposition, and a lack of a fibrotic capsule.

### Extended delivery of rPN increases cardiomyocyte cell cycle activity and angiogenesis in the infarct border zone

**Figure 6:**
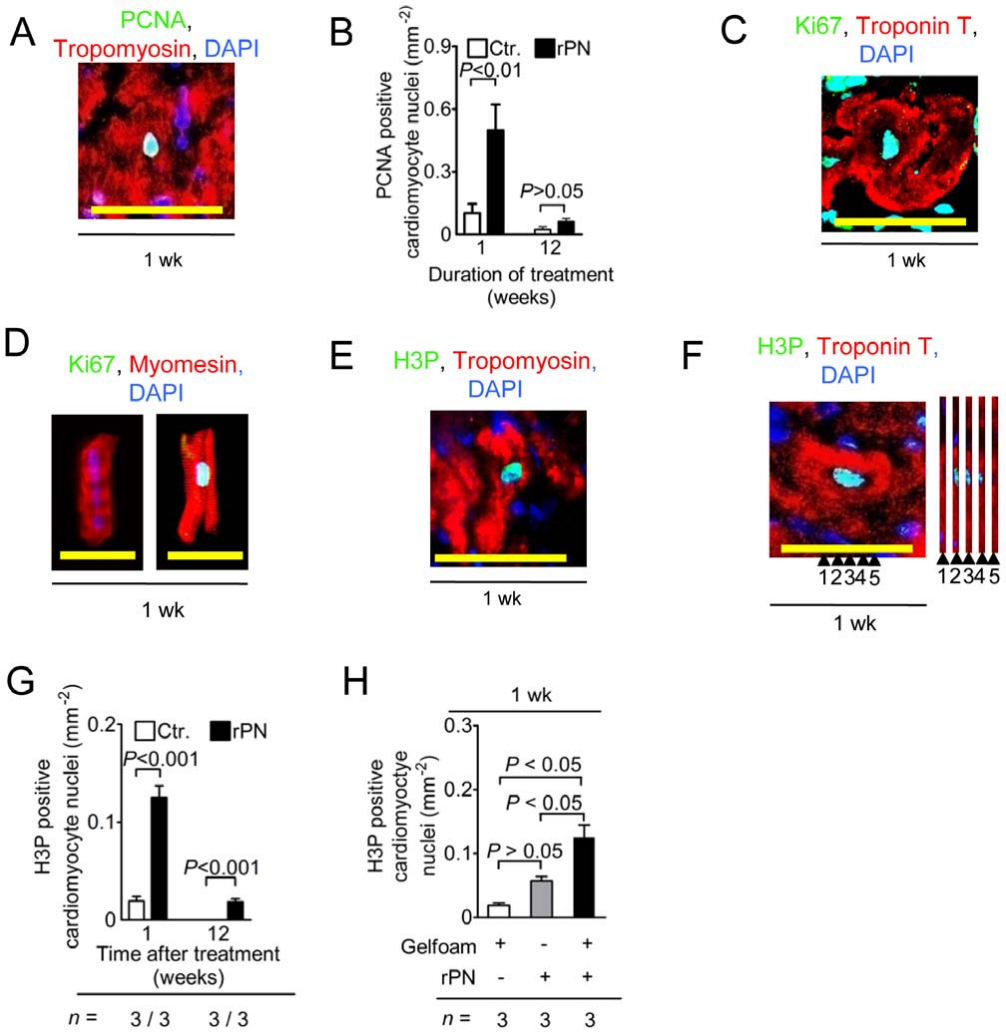
Gelfoam loaded with rPN increases cardiomyocyte cell cycle activity after MI. (A–D) Visualization and quantification of cell cycle activity in tissue sections and in isolated cardiomyocytes from animals treated with rPN, respectively. (E–F) Determination of cardiomyocyte mitosis by visualization of H3P positive cardiomyocyte nuclei. A series of yz reconstructions is shown to the right of the micrograph. (G) Quantification of cardiomyocyte mitosis after 1 wk and 12 wks of treatment. (H) Quantification of the effect of administering rPN adsorbed onto Gelfoam versus administration of rPN alone on cardiomyocyte mitosis. H3P, phosphorylated histone H3 (S10); Ctr, hearts receiving Gelfoam with PBS; rPN, hearts receiving Gelfoam with periostin peptide; *n* is the number of animals per group. Scale bars, 100 µm.

We have previously demonstrated in rats that rPN increases the cell cycle activity of cardiomyocytes and the degree of vascularization within the border zone after MI [1]. We utilized the known biological activity of rPN to confirm that Gelfoam-induced fibrin hydrogels could provide a sustained release of rPN to the myocardium following intrapericardial administration in a swine model of myocardial infarction. We first visualized the cell cycle activity by staining tissue sections with antibodies against PCNA and Ki67 (**Figure 6A–D**). Quantification revealed that rPN-treated animals had 0.50±0.3 PCNA-positive cardiomyocytes per millimeter squared one week after administration, a 5-fold increase over control animals (*P*<0.01, **Figure 6B**). To verify that the detected cell cycle activity was located in cardiomyocytes, cardiomyocytes were isolated from the border zone by enzymatic digestion and stained with an antibody against Ki67 (**Figure 6D**). Ki67 is a well accepted marker of progression through the cell cycle. As such, we utilized Ki67 to enhance the probability of observing cycling cardiomyocytes given the limited amount of tissue we had for the analysis (as opposed to staining for antibodies against PCNA or H3P which is much more selective for cardiomyocyte progressing through either S- or M-phase, respectively). The results indicated that large rod-shaped cardiomyocytes were in the cell cycle (**Figure 6D**). We then stained tissue sections with an antibody against phosphorylated histone H3 at S10 (H3P) to visualize cells in mitosis (**Figure 6E–F**). Quantification of H3P-positive cardiomyocytes on sections revealed that rPN-treated animals had 0.13±0.01 H3P-positive cardiomyocytes per millimeter squared one week after administration, a 6.5–fold increase over controls (*P*<0.001, **Figure 6G**). Furthermore, our results demonstrate that rPN did not induce cell cycle activity in non-cardiomyocytes (**Figure S2**). To further demonstrate that Gelfoam-induced fibrin hydrogels were critical for the sustained release of rPN to the myocardium, rPN alone was injected into the pericardial space of pigs after myocardial infarction. Quantification of H3P-positive cardiomyocytes on sections revealed that Gelfoam disks loaded with rPN had a 2.2-fold increase in the number of H3P-positive cardiomyocytes compared to pigs injected with rPN alone (*P*<0.05, **Figure 6H**), indicating that Gelfoam is required for a maximal exposure.

**Figure 7:**
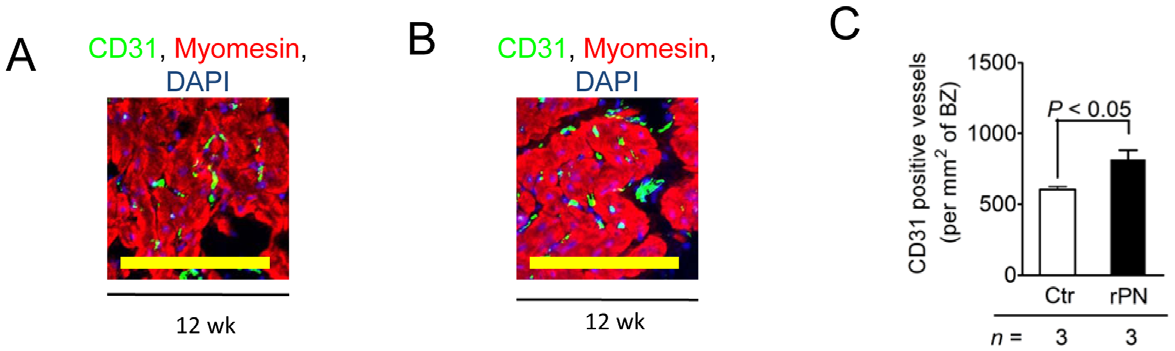
Delivery of rPN/Gelfoam system increased vascularization in the border zone. (A, B) Visualization of CD31 positive vessels within the border zone in both control (A) and rPN (B) treated animals. (C) Quantification of vessel density in the border zone. Ctr, hearts receiving Gelfoam with PBS; rPN, hearts receiving Gelfoam with periostin peptide; *n* is the number of animals per group. Scale bars, 100 µm.

The degree of vascularization, twelve weeks after MI, was visualized by staining tissue sections with antibodies against CD31 to identify the endothelial layer of blood vessels and with myomesin to demarcate cardiomyocytes (**Figure 7A–B**). Quantification revealed a higher number of CD31 positive vessels within the border zone in rPN-treated animals relative to controls (**Figure 7C**, *P*<0.05). Collectively, these data demonstrate that Gelfoam delivered to the pericardial space are useful vehicles for the sustained release of rPN to the myocardium after MI.

### rPN does not alter the fibrotic or inflammatory response

The periostin gene plays a role in the recruitment of fibroblasts to the MI region and in the formation of scar tissue [8], [9]. To evaluate if rPN had an effect on myocardial fibrosis we stained tissue sections from the MI border zone twelve weeks after administration of therapy with acid fuchsin orange-G (AFOG, **Figure S3**). The degree of fibrosis was quantified using two independent techniques. First, we imaged the sections using light microscopy followed by thresholding of the fibrotic areas (stained blue) relative to the myocardial area (stained deep red), which showed no difference between the groups (**Figure S3B**). Second, a pathologist (R.P.) scored the degree of fibrosis on AFOG-stained sections in a blinded manner, using a semi-quantitative scale and found it to be similar between groups (**Figures S3C**). To further investigate the possibility that rPN administration may have an effect on fibroblast proliferation, we quantified the number of non-myocytes undergoing mitosis in the border zone after one week of treatment (**Figure S2B**). rPN did not affect mitotic activity of non-myocytes, consistent with recent findings in periostin knockout mice [28]. The results of these two analyses of fibrosis demonstrate that administration of rPN peptide does not alter myocardial fibrosis after MI, which is in agreement with our previous observations [1] and other studies [8], [9], [28].

The periostin gene has also been reported to influence reactive inflammation [29]–[31]. Thus, we assessed if administration of rPN has an effect on myocardial inflammation. We performed histological analyses at one week after treatment with either rPN or control. The degree of inflammation within the MI border zone was determined in a blinded manner on a semi-quantitative scale by a pathologist (R.P.). The results showed no difference in the degree of inflammation between control pigs and pigs treated with rPN peptide (P<0.05, **Figure S3D**).

## Discussion

Identification of novel factors that regulate myocardial regeneration is an exciting new paradigm in cardiac biology [5]. Our laboratory has recently identified two extracellular factors that have the ability to enhance cardiac function when administered after MI [1], [3]. Given the long time course required for myocardial regeneration to occur, most preclinical interventions employ repeated factor delivery to maintain the therapeutically required dose [1], [3], [32]–[35]. However, this is not clinically practical and often results in poor myocardial uptake and high systemic recirculation. We hypothesized that the translational and clinical potential of pharmaceutical-based therapies would be greatly enhanced by intrapericardial administration of FDA-approved drug-eluting biomaterials. Intrapericardial administration of growth factors alone was previously shown to increase myocardial retention while minimizing uptake by non-target organs [13]. We hypothesized that encapsulation of factors within hydrogels would potentially enable the prolonged release of factors to the myocardium, while further enhancing the retention and bioactivity of rPN, by minimizing lymphatic drainage and proteolytic degradation within the pericardial space.

We chose to encapsulate rPN within Gelfoam disks for several reasons. First, these devices are FDA approved, which greatly enhances the clinical applicability of the proposed method. In fact, a recent pre-clinical study has shown that intrapericardial administration of Gelfoam into the pericardial space of pigs is both feasible and safe [16]. Second, Gelfoam is able to absorb and hold large amounts of fluid within its pores, which provides a simple and efficient method for loading the rPN prior to implantation. Also, Gelfoam disks have been used previously as drug delivery vehicles. It is interesting to note that studies have shown that the incorporation of retardants such as poly(ethylene) glycol or collagen are required to prolong the release of drugs, such as pilocarpine and basic fibroblast growth factor (bFGF), from Gelfoam disks [17], [22]. *In vivo* implantation of Gelfoam is known to induce the formation of fibrin-rich matrices without triggering significant inflammatory or fibrotic responses. We hypothesized that this spontaneous *in vivo* deposition of fibrin within the interstitial sites of Gelfoam could be used as a natural retardant to prolong rPN release, increase rPN bioactivity, and decrease non-specific uptake of rPN by non-target organs.

To examine the role of fibrin deposition on the release kinetics of rPN from Gelfoam disks, we performed both *in vitro* and *in vivo* drug release experiments. The *in vitro* release profile of rPN from Gelfoam disks alone was very rapid compared to rPN release from Gelfoam-fibrin composite hydrogels. These results suggest that fibrin deposition within the interstitial sites of Gelfoam disks is able to effectively modulate rPN release. We performed similar *in vivo* release experiments within the peritoneum of mice. Peritoneal and pericardial fluids have similar compositions, as both are ultra filtrates of the plasma [36]. The *in vivo* release profile of rPN from Gelfoam was much slower than its *in vitro* release from Gelfoam alone. To determine if fibrin deposition played a critical role in modulating the release kinetics, we implanted Gelfoam disks loaded with heparin and rPN. Heparin is known to inhibit fibrin formation by activation of antithrombin and factor Xa [37]. rPN release from heparin-treated Gelfoam disks was rapid, with approximately 100% release occurring within 1 hr of implantation. These data strongly suggest that the fibrin network formation triggered by *in vivo* administration of Gelfoam is critical for the extended release of rPN from these devices.

The *in vivo* applicability of our approach was tested by injecting our delivery systems into the pericardial space of pigs two days after MI. Histological analysis one week after injection revealed minimal inflammation, no evidence of a fibrotic capsule, and extensive fibrin(ogen) and fibrin deposition on the surface of the Gelfoam as well as within the interstitial sites, respectively. These results are similar to those observed from Gelfoam disks explanted from the peritoneal cavity of mice, and suggests that Gelfoam-induced fibrin hydrogels are present after intrapericardial administration of Gelfoam disks. We quantified PCNA and H3P-positive cardiomyocytes as well as CD31-positive vessels in order to characterize the bioactivity and the *in vivo* release of rPN from Gelfoam-induced fibrin hydrogels. Our results clearly indicate that Gelfoam-induced fibrin hydrogels provide a sustained release of rPN for an extended period of time. This correlates with our *in vivo* release profile, in which rPN was shown to be released from the Gelfoam-induced fibrin hydrogels over a period of seven days. This data also suggests that Gelfoam disks injected into the pericardial space are not degraded at an accelerated rate (i.e. they are present 7 days after implantation). It is also important to note that the therapeutic effect of rPN encapsulated within Gelfoam-induced fibrin hydrogels is superior to rPN alone. Injection of rPN into the pericardial space is most likely eliminated by either lymphatic drainage and/or proteolytic cleavage; whereas encapsulation within the Gelfoam hydrogels retains the drug within the cavity and may preserve its bioactivity [14].

Previous studies have shown that the effects of intrapericardial administration are limited to the epicardial surface of the heart [13]. While this did not represent a limitation in the current study, it may be a limiting factor for certain conditions, such as endocardial infarctions, which require the therapeutic to diffuse through the bulk of the myocardium. Also, the results from this study do not permit one to draw conclusions about rPN's ability to stimulate cardiomyocyte proliferation or its effect on cardiac function. Swine cardiomyocytes are known to have multiple nuclei (up to 32 per cardiomyocyte) and stimulation of cell cycle activity does not necessarily correlate with cardiomyocyte division.

Intrapericardial administration of drug eluting biomaterials is a novel, clinically attractive, and effective method for the targeted delivery of therapeutics to the myocardium after MI. We utilized the body's innate immune response (i.e. thrombus formation) to Gelfoam implantation to facilitate the prolonged release of rPN to the myocardium. The approach presented here utilizes established clinical procedures and FDA approved medical devices to enhance factor retention and bioactivity within the pericardial cavity and to prolong the biological effect *in vivo*. This strategy may serve as a platform for rapid testing of new pharmaceuticals that have to be targeted to the heart. In addition, our findings may inspire the future development of advanced biomaterials that enable, for example, the spatio-temporal delivery of multiple factors over various time scales to mediate cardiac injury and to induce myocardial regeneration.

## Materials and Methods

### Ethics Statement

All experiments in this study were conducted in accordance with the guidelines established by the Institutional Animal Care and Use Committee at Mount Sinai School of Medicine and Children's Hospital Boston and the American Association for Accreditation of Laboratory Animal Care and were approved by the Institutional Animal Care and Use Committee at MSSM and CHB. All efforts were made to minimize any pain and suffering felt by the animals.

### In vitro release of rPN-AF488 from Gelfoam hydrogels

Gelfoam (Pfizer) was cut into disks using a standard 6 mm biopsy punch (6 mm×2 mm). Loading was accomplished by application of an aliquot of rPN-AF488 (∼2 µg) to each Gelfoam disk followed by incubation at 4°C for 12 hrs. The volume added to each Gelfoam disk was much less than the maximal absorbable volume to ensure that the applied dose was completely loaded within each gel. Loading was determined by quantifying the amount of fluorescence in solution after degradation of at least 3 individual disks by enzymatic treatment (trypsin). For release studies, the Gelfoam disks, loaded with or without rPN-AF488, were placed within the wells of a 96-well plate containing 50 µL of phosphate-buffered saline (PBS). The plate was covered and placed at 4°C with intermittent agitation. To determine the fraction of rPN-AF488 released from the Gelfoam gels, the PBS within each well was removed at various time points and replaced with an equivalent volume of fresh buffer. The collected buffer was then analyzed on a fluorescent plate reader (λ_ex_ = 494 nm, λ_em_ = 519 nm), and the amount (in µg) of rPN-AF488 released was calculated by interpolation from a standard fluorescent calibration curve. At the end of the experiment, all Gelfoam disks were degraded and the amount of retained rPN-AF488 was determined. The cumulative release was calculated by summing the amount of rPN-AF488 released over the various time intervals. The percentage of cumulative rPN-AF488 released is reported as the ratio of the cumulative release of rPN-AF488 (in µg) at any point in time to the initial amount of rPN-AF488 (in µg) that had been loaded.

Gelfoam disks encapsulated within fibrin gels were prepared as follows. Gelfoam disks (6 mm×2 mm) were pre-swollen in a stock solution of thrombin (25 U/mL) for 30 mins at room temperature. The disks were subsequently allowed to dry at 4°C for 3 days. rPN-AF488 (∼2 µg) was loaded into individual thrombin-loaded Gelfoam disks by incubation at RT for 30 min. Again, the volumes added were much smaller than the maximum swelling volume, which ensured that the applied dose was completely loaded within each gel. The thrombin/rPN-AF488 loaded Gelfoam disks were then placed into a solution of fibrinogen (25 µL, 3.3 mg/mL solution) for 20 mins at 37°C. The amount of rPN-AF488 loaded was determined by enzymatic degradation of at least 3 Gelfoam-fibrin hydrogels. The percentage release of rPN-AF488 was determined as described above.

### In Vivo release of rPN-AF488 from Gelfoam hydrogels

Gelfoam disks (6 mm×2 mm) were pre-swollen in a stock solution of heparin (5000 U/mL) for 30 min at room temperature. The disks were subsequently allowed to dry at 4°C for 3 days. rPN-AF488 (∼2 µg) was allowed to absorb into Gelfoam disks, loaded with or without heparin, for 30 min at room temperature prior to implantation. The Gelfoam disks were subsequently implanted into the peritoneal cavity of healthy adult mice (1 disk/animal; 3 animals per time point). Gelfoam disks loaded with heparin and rPN-AF488 were extracted after 30 mins, 1 hr, and 2 hrs; whereas Gelfoam disks loaded with rPN-AF488 only were explanted after 30 mins, 1 hr, 20 hrs, 48 hrs, 96 hrs, and 168 hrs. The animals were euthanized, the disks retrieved, and they were degraded in Eppendorf vials via treatment with trypsin (25 µL, 3 mg/ml, 37°C for 20 min). Vials were spun down at 14,000×g, the supernatant was added to a black, 96-well flat bottom plate, and the fluorescence was quantified using a standard fluorescent plate reader. The amount of rPN-AF488 loaded was determined by enzymatic degradation of at least 3 Gelfoam-heparin disks (not implanted). The amount of rPN-AF488 retained within the disk (µg) was determined as described above. The percent rPN-AF488 released was calculated according to the following equation:
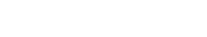
where *X_f_* is the amount of rPN-AF488 remaining in the Gelfoam disk at time *t* and *X_i_* is the amount of rPN-AF488 initially loaded.

### Diffusion of rPN-AF488 into the myocardium

Diffusion of rPN into the myocardium was evaluated ex vivo using fluorescently-labeled rPN. Adult mouse hearts (CB57/SL6) were isolated using standard techniques and immediately placed in ice cold PBS to flush out the ventricles. Hearts were glued to the bottom of Petri dishes (35 mm) with the left ventricle pointing up. Hearts then received no injury/Gelfoam/rPN-AF488 (control), cryo injury/Gelfoam/rPN-AF488 (3 independent 5 second exposures over the same region using a 3 mm probe) or cryo injury only (same exposure). Gelfoam sponges loaded with rPN-AF488 (0.84 µg) were glued to the surface of the heart. In the case of the cryo-injured hearts, care was taken to ensure that the Gelfoam was directly over the injured area. Culture media (containing 10% FBS) was then added to the Petri dishes and the plates incubated at 37°C in 5% CO2 for 2 hours. The hearts were then carefully released from the dishes using a razor blade, rinsed in ice-cold PBS, and immediately embedded in OCT. Detailed information on the histological analysis and quantification of rPN-488 diffusion is provided in the File S1.

### Myocardial infarction (MI) model, delivery of rPN, and sample collection

All manipulations were approved by the IACUC and performed at Mount Sinai School of Medicine (MSSM). In order to enhance the clinical applicability of this delivery system, Gelfoam disks were milled using a standard bone rasp, which enabled administration via injection through standard catheters [16]. Myocardial infarctions (MI) were created in 20 kg female Yorkshire pigs by placement of a platinum coil within the left anterior descending coronary artery (MSSM). Two days after infarct generation, the cardiac function was assessed (i.e. ejection fraction, peak ejection rate, peak filling rate, cardiac index, and dPdt/P) and animals that had similar values (i.e. within 20%) were used in the study. The animals were randomly assigned to control (6 animals) or test groups (7 animals). Animals underwent a thoracotomy to expose the pericardium. Gelfoam disks loaded with either saline (7 mL; control group) or rPN (0.1 mg/mL; 0.7 mg per pig; treatment group) were injected into the pericardial cavity (**Figure 4B**). The pericardium was sutured to prevent leakage into the thoracic cavity. Pigs were euthanized one and twelve weeks after implantation of the delivery system. Samples from the MI border zone, from within the MI, and from the non-infarcted inferior wall were sectioned into 3 zones (epicardium, myocardium, and endocardium), snap frozen or placed in OCT and subsequently shipped to Children's Hospital Boston.

### Immunofluorescence

Heart samples were embedded in optimal cutting temperature compound (OCT) and placed at −80°C. We prepared cryosections (14 µm thick), fixed them in 10% buffered formalin, permeabilized with 0.5% NP-40 dissolved in phosphate buffered saline (PBS), and blocked with goat serum (5%, Sigma). Sections were subsequently treated with primary antibodies against PCNA (Abcam), Ki67 (Abcam) and phosphorylated histone-3 (S10, Millipore) to visualize cardiomyocytes in the cell cycle, troponin T, tropomyosin, and myomesin (Abcam) to visualize cardiomyocyte-specific proteins in the contractile apparatus, CD31 (Abcam) to visualize the presence of blood vessels, and fibrinogen to visualize the presence of fibrin/fibrinogen (Ventana Medical Systems) within Gelfoam disks. Sections were subsequently treated with secondary antibodies conjugated to either Alexa 488 or 594. Nuclei were stained with DAPI. To quantify the number of PCNA and H3P positive cardiomyocyte nuclei per millimeter squared of myocardium we analyzed 3 different hearts per group. For each heart we randomly selected and counted the number of PCNA or H3P positive cardiomyocyte events from 6 slides containing 3–4 sections per slide (only 1 section/slide was quantified). The area of each tissue section was determined using the method of point counts [38] and the ratio of PCNA or H3P positive cardiomyocyte nuclei per area of myocardium was calculated. Quantification of the number of CD31 positive blood vessels was accomplished by acquiring multiple (∼10–15) fluorescent images from each tissue section, using a random systematic approach. The number of vessels per image was quantified using ImageJ and divided by the area of the field of view. Images were acquired using an Olympus IX-81 epifluorescence microscope with LUCPLFL 40× and UPLFL 10× lenses equipped with a Hamamatsu CM CCD camera. Quantification of PCNA and H3P data was independently validated by BDP and SA.

### Histology

Gelfoam disks explanted from the peritoneal cavity of mice were harvested and immediately fixed in 3.7% formaldehyde overnight. Samples were either frozen at −80°C or embedded in optimal cutting temperature. Gelfoam disks were cut into 10 µm sections and re-fixed in 10% buffered formalin. Gelfoam disks explanted from the pericardial cavity of pigs were harvested and either embedded in OCT compound or snap frozen in liquid nitrogen. Samples were cut into 14 µm sections and fixed in 10% buffered formalin. Sections were stained with hematoxylin and eosin to visualize inflammation and acid fuchsin orange-G (AFOG) to visualize fibrin deposition. Images were acquired using a Zeiss Axioplan 2 epifluorescence microscope with Plan-Neofluar 10× and 40× lenses equipped with a Zeiss Axiocam CCD camera.

### Isolation of cardiomyocytes

Frozen samples of myocardial tissue were brought to room temperature and immediately fixed in 10% buffered formalin. The tissue was subsequently incubated with collagenase D (2.4 mg/mL, Roche) and B (1.8 mg/mL, Roche) for 24 hours at 37°C with constant agitation. The supernatant was collected and spun down to yield the isolated cardiomyocytes. This procedure was repeated until no more cardiomyocytes were dissociated from the tissue. The cardiomyocytes were stained for the presence of cell cycle markers as described in the Immunofluorescence section. The efficiency of the isolation procedure was 91.3±3% (by mass) and the percent intactness was 92.4±0.4% (determined by cadherin staining). The percents are given as mean ± SEM, *n* = 8 hearts.

### Statistical Analysis

One-way ANOVA was used to analyze myocardial fibrosis and cell cycle data. Two-tailed Student's t-test was used for vascular density and non-cardiomyocyte proliferation analyses. All measurements were reported as the mean ± SEM. Significance was accepted at p<0.05. Number of animals in pig experiments: 6 control (Gelfoam only) and 7 treatment (Gelfoam/rPN). Number of animals in mouse experiments: 3 control (Gelfoam only) and 3 treatment (Gelfoam/rPN-AF488).

## Supporting Information

File S1 Supplemental information is available for the following: synthesis and characterization of rPN-AF488, quantification of non-myocyte cell cycle activity, and quantification of myocardial fibrosis and inflammation.
(DOCX)

Figure S1 **Synthesis and characterization of rPN-AF488.** (A) Schematic comparing the protein structure of the type 1 variant of human periostin with the recombinant truncated peptide used in this study. The recombinant PN peptide is truncated from amino acid 1–22 and from 672–836. (B) Schematic representation of the bio-conjugation technique used to fluorescently-label rPN with Alexa Fluor 488. (C) SDS-PAGE analysis of rPN-AF488 and rPN (control).
(TIF)

Figure S2 **Administration of Gelfoam loaded with rPN does not stimulate non-cardiomyocyte cell cycle activity** (A) Photomicrograph of a H3P positive non-cardiomyocyte within the infarct border zone. (B) Quantification of non-cardiomyocyte mitosis after 1 wk of treatment. Ctr., control hearts receiving Gelfoam with buffer; rPN, hearts receiving Gelfoam with periostin peptide; *n* is the number of animals per group. Scale bars, 100 µm.
(TIF)

Figure S3 **Delivery of Gelfoam loaded with rPN does not alter myocardial fibrosis or inflammation in the infarct border zone.** (A–C) Visualization and quantification of myocardial fibrosis from tissue sections stained with acid fuchsin orange G (AFOG). Fibrosis was determined both quantitatively using an image analysis software (B) and semi-quantitatively by a pathologist in a blinded manner (C). (D) Visualization of myocardial inflammation using Hematoxylin and eosin staining (H&E). (E) Quantification of myocardial inflammation was performed in a blinded manner by a pathologist. Ctr., control hearts receiving Gelfoam with buffer; rPN, hearts receiving Gelfoam with periostin peptide; *n* is the number of animals per group. Scale bars, 100 µm.
(TIF)

## References

[1] Kühn B, del Monte F, Hajjar RJ, Chang Y-S, Lebeche D (2007). Periostin induces proliferation of differentiated cardiomyocytes and promotes cardiac repair.. Nature medicine.

[2] Abraham WT, Hasan A, Poole-Wilson P, Fuster V, O'Rourke RA, Walsh RA, Poole-Wilson P (2008). Diagnosis and Management of Heart Faliure.. Hurst's The Heart 12th ed.

[3] Bersell K, Arab S, Haring B, Kuhn B (2009). Neuregulin1/ErbB4 signaling induces cardiomyocyte proliferation and repair of heart injury.. Cell.

[4] Ruvinov E, Leor J, Cohen S (2010). The promotion of myocardial repair by the sequential delivery of IGF-1 and HGF from an injectable alginate biomaterial in a model of acute myocardial infarction.. Biomaterials.

[5] Segers VF, Lee RT (2010). Protein therapeutics for cardiac regeneration after myocardial infarction.. J Cardiovasc Transl Res.

[6] Norris RA, Moreno-Rodriguez R, Hoffman S, Markwald RR (2009). The many facets of the matricelluar protein periostin during cardiac development, remodeling, and pathophysiology.. J Cell Commun Signal.

[7] Johnatty SE, Dyck JR, Michael LH, Olson EN, Abdellatif M (2000). Identification of genes regulated during mechanical load-induced cardiac hypertrophy.. J Mol Cell Cardiol.

[8] Oka T, Xu J, Kaiser Ra, Melendez J, Hambleton M (2007). Genetic manipulation of periostin expression reveals a role in cardiac hypertrophy and ventricular remodeling.. Circulation research.

[9] Shimazaki M, Nakamura K, Kii I, Kashima T, Amizuka N (2008). Periostin is essential for cardiac healing after acute myocardial infarction.. The Journal of experimental medicine.

[10] Snider P, Hinton RB, Moreno-Rodriguez RA, Wang J, Rogers R (2008). Periostin is required for maturation and extracellular matrix stabilization of noncardiomyocyte lineages of the heart.. Circ Res.

[11] Stanton LW, Garrard LJ, Damm D, Garrick BL, Lam A (2000). Altered patterns of gene expression in response to myocardial infarction.. Circ Res.

[12] Wang D, Oparil S, Feng JA, Li P, Perry G (2003). Effects of pressure overload on extracellular matrix expression in the heart of the atrial natriuretic peptide-null mouse.. Hypertension.

[13] Laham RJ, Rezaee M, Post M, Xu X, Sellke FW (2003). Intrapericardial administration of basic fibroblast growth factor: myocardial and tissue distribution and comparison with intracoronary and intravenous administration.. Catheter Cardiovasc Interv.

[14] Lin CC, Anseth KS (2009). PEG hydrogels for the controlled release of biomolecules in regenerative medicine.. Pharm Res.

[15] Hamalainen KM, Maatta E, Piirainen H, Marianne S, Vaisanen A (1998). Roles of acid/base nature and molecular weight in drug release from matrices of gelfoam and monoisopropyl ester of poly(vinyl methyl ether-maleic anhydride).. J Control Release.

[16] Ladage D, Turnbull IC, Ishikawa K, Takewa Y, Rapti K (2011). Delivery of gelfoam-enabled cells and vectors into the pericardial space using a percutaneous approach in a porcine model.. Gene Ther.

[17] Nadkarni SR, Yalkowsky SH (1993). Controlled delivery of pilocarpine. 1. In vitro characterization of Gelfoam matrices.. Pharm Res.

[18] Simamora P, Nadkarni SR, Lee Y-C, Yalkowsky SH (1998). Controlled delivery of pilocarpine. 2. In-vivo evaluation of Gelfoam device.. International journal of Pharmaceutics.

[19] Lee YC, Simamora P, Yalkowsky SH (1997). Effect of Brij-78 on systemic delivery of insulin from an ocular device.. J Pharm Sci.

[20] Simamora P, Lee YC, Yalkowsky SH (1996). Ocular device for the controlled systemic delivery of insulin.. J Pharm Sci.

[21] Lee YC, Simamora P, Yalkowsky SH (1997). Systemic delivery of insulin via an enhancer-free ocular device.. J Pharm Sci.

[22] Song S, Morawiecki A, USPTO, editor (1995). Collagen-containing sponges as drug delivery compositions for proteins.. United States of America: Amgen Inc.

[23] Barbolt TA, Odin M, Leger M, Kangas L (2001). Pre-clinical subdural tissue reaction and absorption study of absorbable hemostatic devices.. Neurol Res.

[24] Arand AG, Sawaya R (1986). Intraoperative chemical hemostasis in neurosurgery.. Neurosurgery.

[25] Brem H, Kader A, Epstein JI, Tamargo RJ, Domb A (1989). Biocompatibility of a biodegradable, controlled-release polymer in the rabbit brain.. Sel Cancer Ther.

[26] Tamargo RJ, Epstein JI, Reinhard CS, Chasin M, Brem H (1989). Brain biocompatibility of a biodegradable, controlled-release polymer in rats.. J Biomed Mater Res.

[27] Kobayashi H, Minatoguchi S, Yasuda S, Bao N, Kawamura I (2008). Post-infarct treatment with an erythropoietin-gelatin hydrogel drug delivery system for cardiac repair.. Cardiovasc Res.

[28] Teekakirikul P, Eminaga S, Toka O, Alcalai R, Wang L (2010). Cardiac fibrosis in mice with hypertrophic cardiomyopathy is mediated by non-myocyte proliferation and requires Tgf-beta.. J Clin Invest.

[29] Blanchard C, Mingler MK, McBride M, Putnam PE, Collins MH (2008). Periostin facilitates eosinophil tissue infiltration in allergic lung and esophageal responses.. Mucosal Immunol.

[30] Sidhu SS, Yuan S, Innes AL, Kerr S, Woodruff PG (2010). Roles of epithelial cell-derived periostin in TGF-beta activation, collagen production, and collagen gel elasticity in asthma.. Proc Natl Acad Sci U S A.

[31] Woodruff PG, Boushey HA, Dolganov GM, Barker CS, Yang YH (2007). Genome-wide profiling identifies epithelial cell genes associated with asthma and with treatment response to corticosteroids.. Proc Natl Acad Sci U S A.

[32] Chan JM, Zhang L, Tong R, Ghosh D, Gao W (2010). Spatiotemporal controlled delivery of nanoparticles to injured vasculature.. Proc Natl Acad Sci U S A.

[33] Engel FB, Hsieh PC, Lee RT, Keating MT (2006). FGF1/p38 MAP kinase inhibitor therapy induces cardiomyocyte mitosis, reduces scarring, and rescues function after myocardial infarction.. Proc Natl Acad Sci U S A.

[34] Scott RC, Wang B, Nallamothu R, Pattillo CB, Perez-Liz G (2007). Targeted delivery of antibody conjugated liposomal drug carriers to rat myocardial infarction.. Biotechnol Bioeng.

[35] Woo YJ, Panlilio CM, Cheng RK, Liao GP, Suarez EE (2007). Myocardial regeneration therapy for ischemic cardiomyopathy with cyclin A2.. J Thorac Cardiovasc Surg.

[36] Maurer FW, Warren MF, Drinker CK (1940). The Composition of Mammalian Pericardial and Peritoneal Fluids.. Am J Physiol.

[37] Hirsh J, Warkentin TE, Shaughnessy SG, Anand SS, Halperin JL (2001). Heparin and low-molecular-weight heparin: mechanisms of action, pharmacokinetics, dosing, monitoring, efficacy, and safety.. Chest.

[38] Howard MA, Roberts N, Garcia-Finana M, Cowell PE (2003). Volume estimation of prefrontal cortical subfields using MRI and stereology.. Brain Res Brain Res Protoc.

