# Supplementary material for: Intrapericardial Delivery of Gelfoam Enables the Targeted Delivery of Periostin Peptide after Myocardial Infarction by Inducing Fibrin Clot Formation
Source: PLoS One. 2012 May 10;7(5):e36788. doi: 10.1371/journal.pone.0036788 (PMC3349650; doi:10.1371/journal.pone.0036788)
Supplement: File S1 — Supplemental information is available for the following: synthesis and characterization of rPN-AF488, quantification of non-myocyte cell cycle activity, and quantification of myocardial fibrosis and inflammation. (DOCX) [file pone.0036788.s001.docx]

## Supplemental Data

## Intrapericardial Delivery of Gelfoam Enables the Targeted Delivery of Periostin Peptide after Myocardial Infarction by Inducing Fibrin Clot Formation

## Brian D. Polizzotti,^1,2^ Shima Arab,^1^ and Bernhard Kühn^1,2^

Supplemental Data for this manuscript include:

Supplemental Experimental Procedures

Figures and Legends S1-S3

## Supplemental Experimental Procedures

**Synthesis and characterization of fluorescently-labeled rPN (rPN-AF488).**

rPN peptide (BioVendor, **Figure S1A**) was conjugated with Alexa Fluor 488 using a modified procedure from a commercially available kit (Invitrogen, **Figure S1B**). rPN peptide (0.1 mg) was dissolved in dimethylsulfoxide (200 μL) containing diisopropylethylamine (10 μL). To this solution was added 1 mg of Alexa Fluor 488-tetrafluorophenyl ester. The reaction was allowed to proceed for 24 hrs at room temperature in the absence of light. The conjugated rPN peptide (rPN-AF488) was purified by dialysis against acetate buffer (0.1 M sodium acetate; pH 4.0) for 2 days at room temperature and freeze-dried to yield the final conjugated product. rPN-AF488 was subsequently dissolved in 200 μL of acetate buffer, the concentration determined by BCA protein analysis, and the purity determined by SDS-PAGE analysis (**Figure S1C**). Serial dilutions of PN-AF488 were prepared, analyzed via fluorescence spectroscopy, and standard fluorescence calibration curves obtained.

**Quantification of non-cardiomyocyte cell cycle activity.**

At Children’s Hospital Boston, we prepared cryosections (14 μm thick), fixed them in 10% buffered formalin, permeabilized with 0.5% NP-40 dissolved in phosphate buffered saline (PBS), and blocked with goat serum (5%, Sigma). Sections were subsequently treated with primary antibodies against phosphorylated histone-3 (S10, Millipore) and troponin I (Abcam) to visualize all non-cardiomyocytes undergoing mitosis. Sections were subsequently treated with secondary antibodies conjugated to either Alexa 488 or 594. Nuclei were stained with DAPI. Quantification of the number of cycling non-cardiomyocytes was accomplished by acquiring multiple (between 5-8) fluorescent images from each tissue section (3 tissue sections/animal; 3 animals/group). The number of cycling non-cardiomyocytes was counted and divided by the area of the field of view (**Figure S2**).

**Histological analysis of fibrosis and inflammation.**

Hearts were harvested, embedded in optimal cutting temperature (OCT) compound, and mailed to Children’s Hospital Boston. The hearts were cut into 14 μm sections and fixed in 10% buffered formalin. We visualized myocardial fibrosis and inflammation in the MI border zone twelve weeks after administration of therapy by staining with acid fuchsin orange-G (AFOG, **Figure S3A-C**) or hemotoxylin and eosin (H&E, **Figure S3D**). The degree of fibrosis was quantified using two independent techniques. First, we imaged the sections using light microscopy followed by thresholding of the fibrotic areas (stained blue) relative to the myocardial area (stained deep red), which showed no difference between the groups (**Figure S3B**). Second, a pathologist (R.P.) scored the degree of fibrosis on AFOG-stained sections in a blinded manner, using a semi-quantitative scale and found it to be similar between groups (**Figure S3C**). The pathologist also scored the degree of inflammation on H&E stained sections in a similar manner. The results showed no difference in inflammation between control and rPN-treated animals.

**Histological analysis of rPN-AF488 diffusion.**

Sections (20 μm) were prepared by cutting perpendicular to the main axis of the heart (imaginary line connecting the base of the heart to the apex). Tissue sections were then immediately visualized using standard fluorescence microscopy (4x, 500ms exposure time, **Figure 3**) or fixed and stained to visualize the myocardium (α-actinin) and rPN (anti-OSF2). However, the fixation and permeabilization steps masked the fluorescence of the rPN-AF488. As such, quantification of the rPN-diffusion was performed on unfixed tissue sections

**Quantification of rPN-AF488 diffusion.**

Fluorescent images were acquired using an Olympus IX-81 epifluorescence microscope with a UPLFL 4x lens equipped with a Hamamatsu CM CCD camera. Images were exported as TIFF files and processed using ImageJ. The inward diffusion of rPN was measured by quantifying the fluorescence intensity. We utilized the Gelfoam to readily identify the epicardial surface of the region of interest (i.e. infarcted region, **Figure 3**). The line tool was then used to quantify the pixel intensity across the region of interest (6 lines per image). To account for non-specific fluorescence, the average pixel intensity from the cryo injured only samples (**Figure 3C**) was subtracted from all the test samples (~10 grey value units). The inward diffusion was defined as the distance at which the grey value was < 10 for 5 or more measurements. This was performed 6 times per image, and the average of these was defined as the inward diffusion.
