# Supplementary figures and images for: Intrapericardial Delivery of Gelfoam Enables the Targeted Delivery of Periostin Peptide after Myocardial Infarction by Inducing Fibrin Clot Formation
Source: PLoS One. 2012 May 10;7(5):e36788. doi: 10.1371/journal.pone.0036788 (PMC3349650; doi:10.1371/journal.pone.0036788)

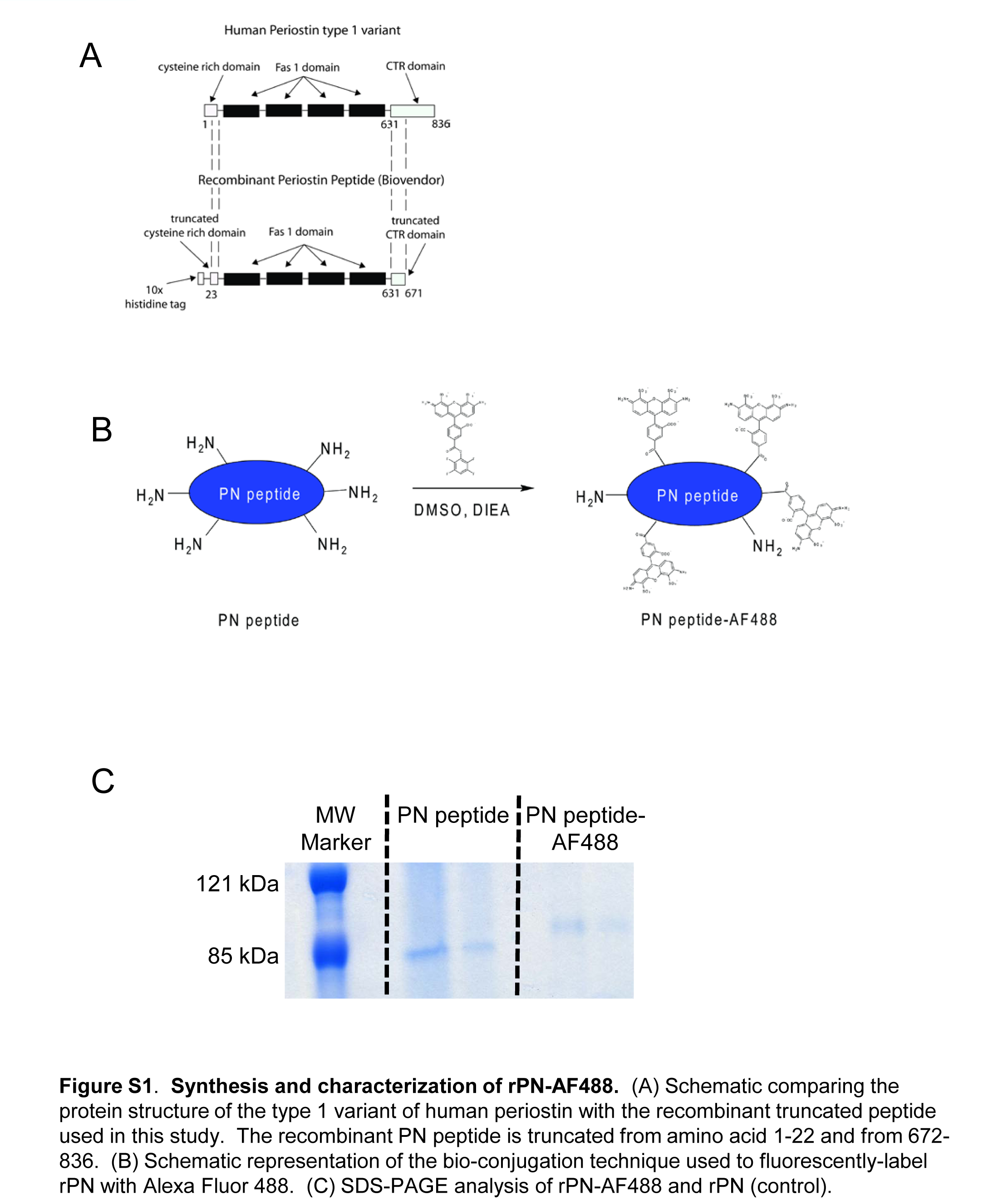

Supplement: Figure S1 — Synthesis and characterization of rPN-AF488. (A) Schematic comparing the protein structure of the type 1 variant of human periostin with the recombinant truncated peptide used in this study. The recombinant PN peptide is truncated from amino acid 1–22 and from 672–836. (B) Schematic representation of the bio-conjugation technique used to fluorescently-label rPN with Alexa Fluor 488. (C) SDS-PAGE analysis of rPN-AF488 and rPN (control). (TIF) [file pone.0036788.s002.tif]

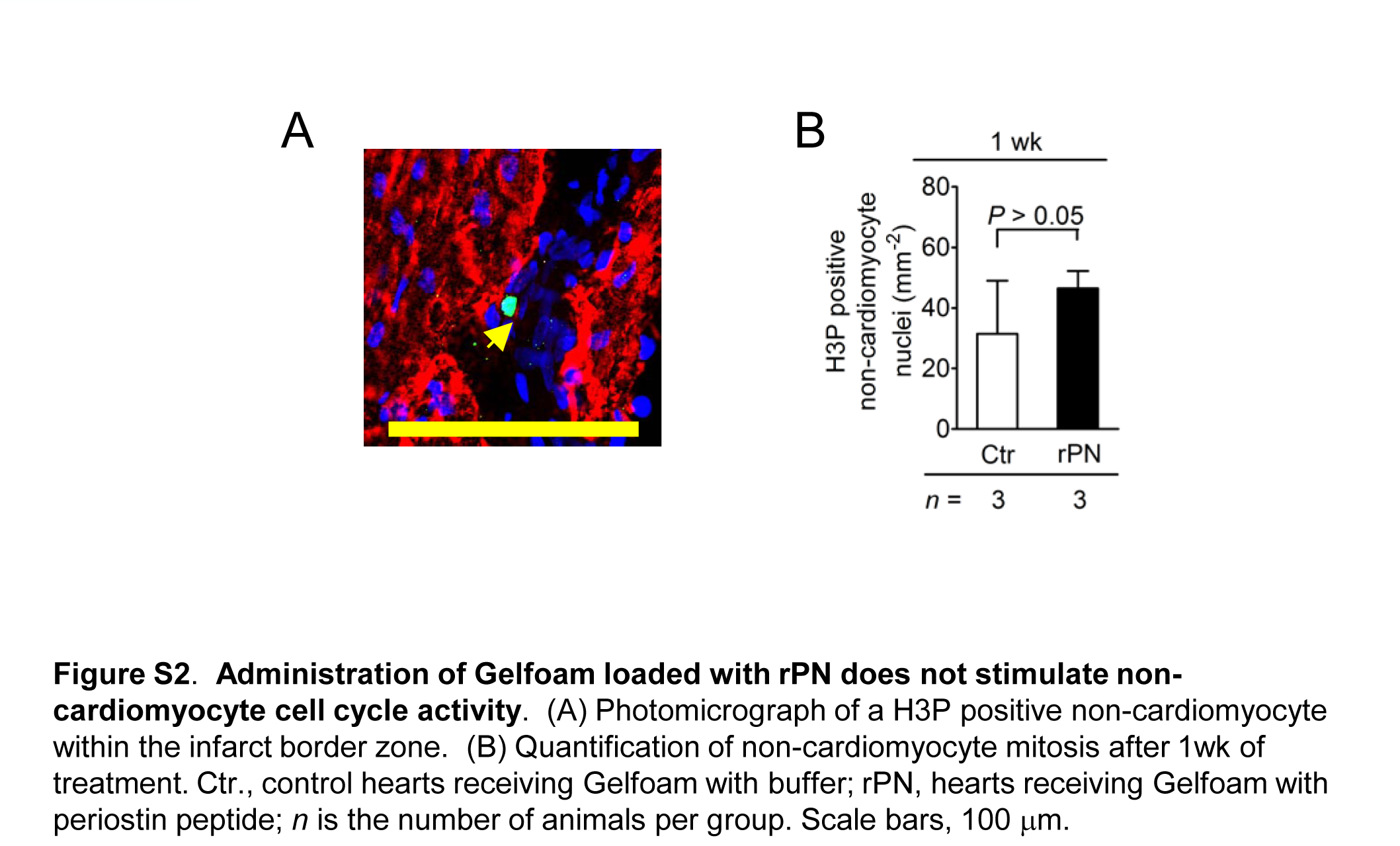

Supplement: Figure S2 — Administration of Gelfoam loaded with rPN does not stimulate non-cardiomyocyte cell cycle activity (A) Photomicrograph of a H3P positive non-cardiomyocyte within the infarct border zone. (B) Quantification of non-cardiomyocyte mitosis after 1 wk of treatment. Ctr., control hearts receiving Gelfoam with buffer; rPN, hearts receiving Gelfoam with periostin peptide; n is the number of animals per group. Scale bars, 100 µm. (TIF) [file pone.0036788.s003.tif]

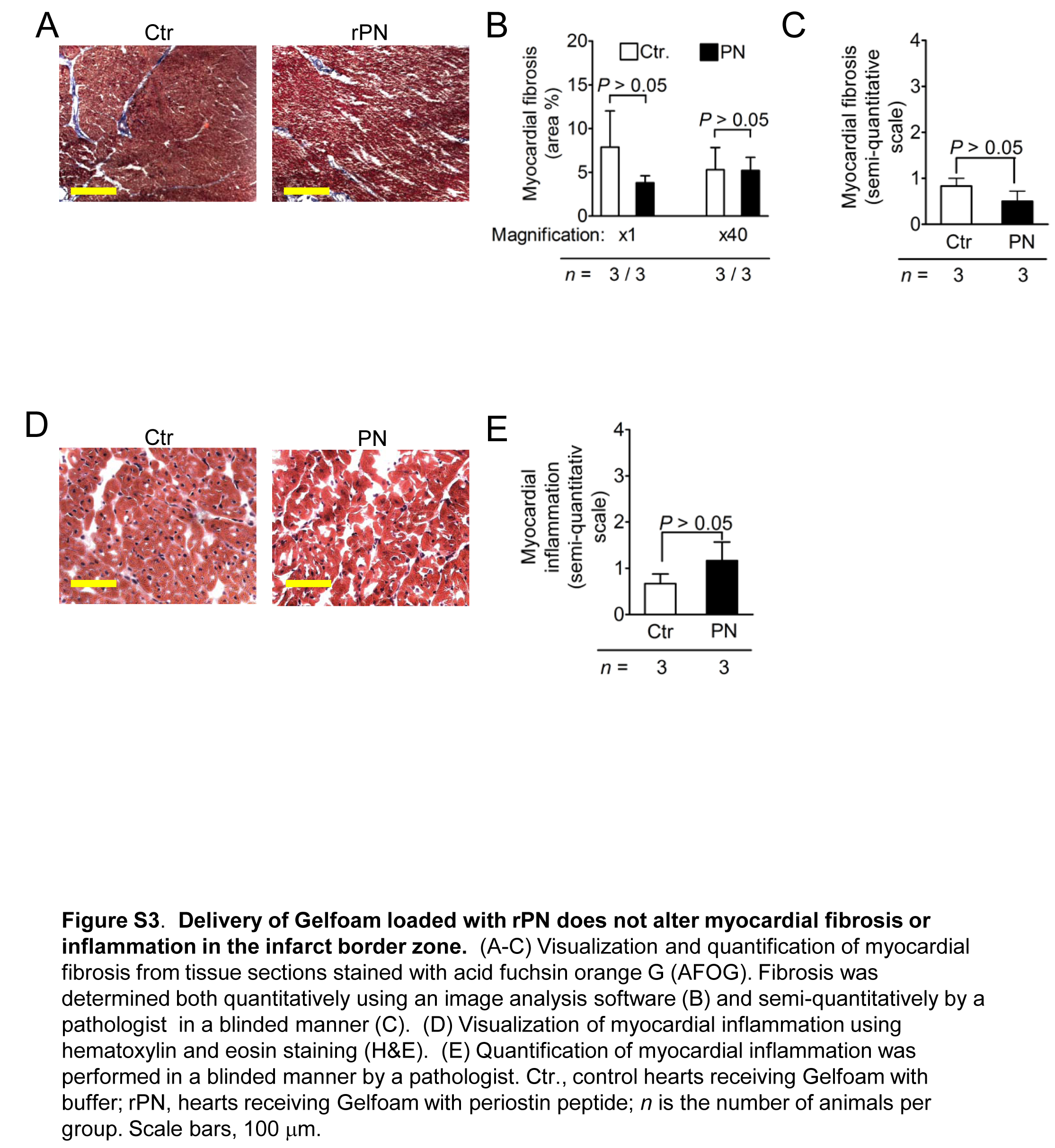

Supplement: Figure S3 — Delivery of Gelfoam loaded with rPN does not alter myocardial fibrosis or inflammation in the infarct border zone. (A–C) Visualization and quantification of myocardial fibrosis from tissue sections stained with acid fuchsin orange G (AFOG). Fibrosis was determined both quantitatively using an image analysis software (B) and semi-quantitatively by a pathologist in a blinded manner (C). (D) Visualization of myocardial inflammation using Hematoxylin and eosin staining (H&E). (E) Quantification of myocardial inflammation was performed in a blinded manner by a pathologist. Ctr., control hearts receiving Gelfoam with buffer; rPN, hearts receiving Gelfoam with periostin peptide; n is the number of animals per group. Scale bars, 100 µm. (TIF) [file pone.0036788.s004.tif]
